# Elevated Src family kinase activity stabilizes E-cadherin-based junctions and collective movement of head and neck squamous cell carcinomas

**DOI:** 10.18632/oncotarget.3071

**Published:** 2014-12-26

**Authors:** Laurence Veracini, Dominique Grall, Sébastien Schaub, Stéphanie Beghelli-de la Forest Divonne, Marie-Christine Etienne-Grimaldi, Gérard Milano, Alexandre Bozec, Emmanuel Babin, Anne Sudaka, Juliette Thariat, Ellen Van Obberghen-Schilling

**Affiliations:** ^1^ University of Nice Sophia Antipolis, UFR Sciences, Nice, France; ^2^ CNRS, UMR7277, Nice, France; ^3^ Inserm, U1091, Nice, France; ^4^ Department of Pathology, Centre Antoine Lacassagne, Nice, France; ^5^ Laboratory of Oncopharmacology, Centre Antoine Lacassagne, Nice, France; ^6^ Department of Otorhinolaryngology, Centre Antoine Lacassagne, Nice, France; ^7^ Department of Otorhinolaryngology and Cervicofacial Surgery, CHU, Caen, France

**Keywords:** Head and neck cancer, Src kinases, E-cadherin, collective migration

## Abstract

EGF receptor (EGFR) overexpression is thought to drive head and neck carcinogenesis however clinical responses to EGFR-targeting agents have been modest and alternate targets are actively sought to improve results. Src family kinases (SFKs), reported to act downstream of EGFR are among the alternative targets for which increased expression or activity in epithelial tumors is commonly associated to the dissolution of E-cadherin-based junctions and acquisition of a mesenchymal-like phenotype. Robust expression of total and activated Src was observed in advanced stage head and neck tumors (N=60) and in head and neck squamous cell carcinoma lines. In cultured cancer cells Src co-localized with E-cadherin in cell-cell junctions and its phosphorylation on Y419 was both constitutive and independent of EGFR activation. Selective inhibition of SFKs with SU6656 delocalized E-cadherin and disrupted cellular junctions without affecting E-cadherin expression and this effect was phenocopied by knockdown of Src or Yes. These findings reveal an EGFR-independent role for SFKs in the maintenance of intercellular junctions, which likely contributes to the cohesive invasion E-cadherin-positive cells in advanced tumors. Further, they highlight the need for a deeper comprehension of molecular pathways that drive collective cell invasion, in absence of mesenchymal transition, in order to combat tumor spread.

## INTRODUCTION

Head and neck squamous cell carcinomas (HNSCC) that arise in the oral cavity, pharynx and larynx represent 3% of all malignancies and the sixth most common malignancy worldwide. In spite of significant improvements in chemo/radiotherapy and surgical techniques, the 5-year survival rate of 50% has not improved for the last few decades [1]. HNSCC tend to escape primary treatment causing significant numbers of patients to develop second primary tumors, loco-regional recurrences and distant metastases. Overexpression of the Epidermal Growth Factor Receptor (EGFR) is one event linked to head and neck carcinogenesis and several EGFR-targeted therapies have been brought to clinical trials or approved in the case of cetuximab plus radiation [2], for the treatment of HNSCC. Although EGFR-targeting agents are well-tolerated, clinical responses remain modest and alternate targets and combined treatments are being sought to improve results [3, 4].

Alternative targets include the Src family of membrane-associated non-receptor tyrosine kinases for which elevated expression, or activity, correlates with tumor progression in many cancers (reviewed in [5, 6]. The Src kinase family is comprised of 8 members, 3 of which (Src, Yes and Fyn) are ubiquitously expressed. Kinase activation occurs through a range of mechanisms, including interactions with activated receptors such as the EGFR [7], that disrupt inhibitory intramolecular interactions which hold Src in a closed configuration. Autophosphorylation at tyrosine 419 (Y419, Y416 in chicken) is required for full activation of Src [5] and the phosphorylation status at this site is commonly used to monitor the active kinase.

Src plays a central role in multiple signaling pathways that regulate cell adhesion, invasion and motility. Activation of the kinase in tumor cells is commonly associated with the destabilization of intercellular adhesion observed during the process of epithelial to mesenchymal transition (EMT). This disruption has been ascribed to several mechanisms including downregulation of E-cadherin expression, increased endocytosis of the protein or disruption of E-cadherin-based contacts through integrin signaling (see [8, 9]). Accordingly, pharmacological inhibition of SFKs has been reported in various tumor cell models to recruit E-cadherin to regions of cell-cell contact and stabilize adherens junctions [10-12]. However, most carcinomas invade surrounding stroma as multicellular groups of cells with fully intact cell-cell adhesions. Indeed, this collective mode of locomotion has been described for oral squamous cell carcinomas [13] and is recognized as an important mechanism of cancer cell invasion and metastasis [14-16].

Here we examined the status of Src in human head and neck tumors, the crosstalk of Src with EGFR signaling, and the consequences of Src inhibition on intercellular adhesion and migration of cultured HNSCC. We report that human head and neck tumors display high levels of constitutively active SFKs. In HNSCC-derived cell lines active SFKs were found to localized to cellular junctions. Selective pharmacological inhibition of SFK signaling, or depletion of Src or Yes in these lines disrupted E-cadherin-based adhesions, without affecting the expression of junctional proteins. This finding suggests that SFKs (Src and Yes) can play a role in stabilising intercellular adhesion in E-cadherin-positive HNSCC and this regulatory function appears to be important for collective strategies of tumor cell invasion.

## RESULTS

### Src expression and activation in human head and neck tumors

**Figure 1:**
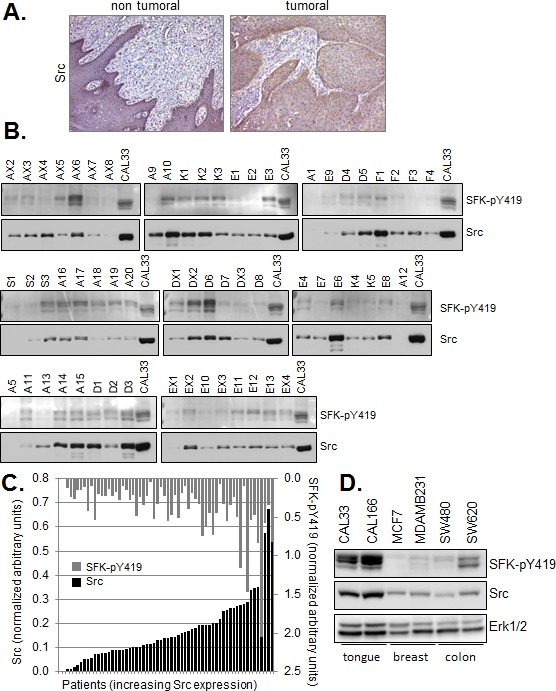
Src and phospho-SFK detection in human tumor samples and cell lines (A) Immunohistochemical staining of Src in a representative formalin fixed paraffin embedded (FFPE) human Squmaous Cell Carcinoma of the tongue. (bar=200μm) (B) Western blot analysis of Src and phospho-SFK (SFK-pY419) in membrane preparations of human head and neck tumor samples (10μg). Controls correspond to membranes (10μg) prepared from exponentially growing CAL33 cells treated for 5 minutes with 30 ng/ml EGF. (C) Quantitative Western blot data were normalized to control values within each series of 6-8 patient samples and plotted with respect to Src expression. Inter-assay variability calculated on control values from six independent runs was <27%. Bars represent single patient samples. (D) Western blot of Src and phospho-SFK (SFK-pY419) in total cell lysates of the indicated human tumor lines.

In human HNSCC, robust Src expression was observed in tumor epithelial cells by immunohistochemistry. Staining in adjacent non-tumoral epithelium was undetectable, or faint and limited to the basal cell layer (Figure 1A). We were unable to assess Src activity in paraffin-embedded human tumors using anti-phospho-Src antibodies therefore we turned to quantitative Western blot analysis in order to determine the status of Src expression and activation in HNSCC. Western blot analysis was performed on membrane preparations from 60 surgically resected human head and neck tumors prior to treatment of patients (Figure 1B). Patients (84% male, median age 58 years old) with histologically proven HNSCC and planned surgery for locally advanced disease with intermediate/poor prognosis (extracapsular spread, > 3 N+ and/or lymphatic or vascular emboli +/− perineural invasion +/− microscopically positive margins +/− pT4 on operative specimen) were enrolled in a French multicenter blinded institutional review board-approved randomized phase II trial of post-operative irradiation with cisplatin ± gefitinib vs placebo. Gefitinib (250mg twice-daily, AstraZeneca Pharmaceuticals) was administered orally for 9.5 weeks concurrently to chemoradiation. Of those enrolled patients, 60 had frozen tumor samples available for Western blot analyses. The phospho-Src antibody used to detect the active form of the kinase recognizes active Src phosphorylated on activation loop residue tyrosine 419 (tyrosine 416 in c-Src of avian origin). This antibody is referred to as SFK-pY419 since in addition to Src, it is able to recognize other Src family kinase (SFK) members. Src and active SFKs were detected in all but two (3.3%) and two (3.3%) tumor samples, respectively. Quantitative chemiluminescence imaging of Western blots revealed a wide range of both total Src and active SFK levels in tumors. The highest expression levels were comparable to or greater than that observed in membranes prepared from a human HNSCC line (CAL33), included as normalization control (Figure 1C). There was a non Gaussian distribution of Src and phospho-SFK expression levels with median levels of 0.139 and 0.269, respectively. A significant correlation was observed between Src expression and phospho-SFK levels in human tumors (Pearson coefficient r=0.494, p≤ 0.001). Neither high expression (above the median) of phospho-SFK or Src was significantly associated in this patient population with overall survival (p=0.83 and 0.33) or disease-free survival defined as time to primary, nodal or metastatic relapse (p= 0.47 and 0.29).

Elevated levels of Src have also been observed in other human malignancies, including breast and colon carcinomas. Importantly, total Src and active SFK expression was found by Western blot analysis to be significantly higher in HNSCC lines (CAL33 and CAL166) than in the commonly used human breast (MCF7, MDAMB231) and colon (SW480, SW620) carcinoma lines (Figure 1D).

### SFK activation is constitutive and independent of EGF receptor activation in HNSCC lines

**Figure 2:**
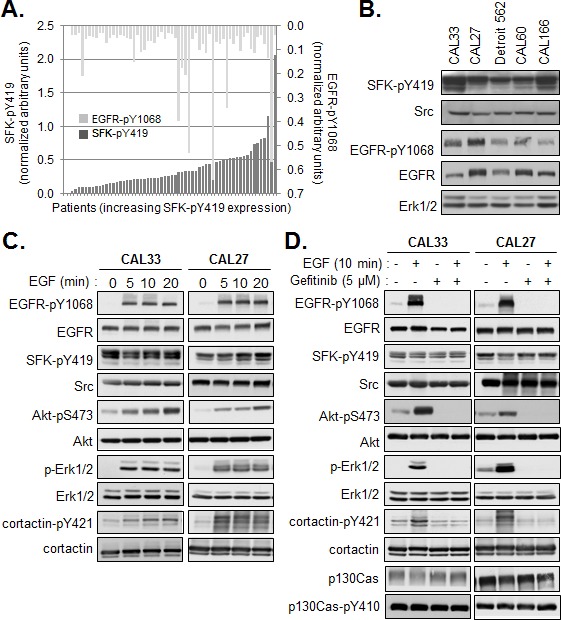
Constitutive activity of SFK is independent of EGFR activation (A) Quantification of phosphorylated SFK (SFK-pY419) and EGFR (EGFR-pY1068) levels determined by Western analysis of membranes from human tumors. Results were normalized to control values within each series of 6-8 patient samples and plotted with respect to phospho-SFK expression (<27% inter-assay variability). Bars represent single patient samples. (B) Western blot of total Src and active SFK and EGFR in lysates prepared from the indicated HNSCC lines. Quantification of phosphorylated SFK (SFK-pY419) and EGFR (EGFR-pY1068) levels determined by Western analysis of membranes from human tumors. (C) Western blot analysis of EGFR and Src signaling pathways in CAL33 and CAL27 cells serum starved for 24h then stimulated for the indicated times with EGF (20ng/ml). (D) Western blot analysis of EGFR and Src signaling pathways in CAL33 and CAL27 cells serum starved for 24h then stimulated or not for 10min with EGF (20ng/ml) in presence or absence of Gefitinib (5μM).

Activation of Src is frequently positioned downstream of EGFR activation in normal and transformed cells, including cells of HNSCC origin [17, 18]. However, the correlation between active SFK levels and active EGFR in our cohort of human tumors, as measured by Western blotting using antibodies directed against the EGFR phosphorylated on tyrosine 1068 (EGFR-pY1068) was quite weak (Figure 2A) (Pearson coefficient r=0.255, p≤ 0.046). Further, we did not observe equivalent levels of the active kinases in a set of exponentially growing HNSCC lines (Figure 2B). Therefore, we examined the effect of EGFR activation on SFK phosphorylation more closely using 2 HNSCC lines, CAL33 and CAL27. As shown in Figure 2C and D, addition of EGF to serum-starved cells stimulated the phosphorylation of EGFR and downstream signaling components including Akt, Erk, without affecting SFK phosphorylation. Conversely, inhibition of the EGFR tyrosine kinase with gefinitib had no effect on active SFK levels under conditions in which it abrogated EGFR, AKT and ERK phosphorylation (Figure 2D). SFK activation was found to be not only independent of EGFR activation in these cells, but also independent of serum growth factors. In contrast to EGFR, Akt and Erk phosphorylation, serum removal had no effect on phospho-SFK levels (Figure 2C, D). Phosphorylation of cortactin on tyrosine 421, a known Src phosphorylation site, was stimulated in response to EGF yet basal phosphorylation, likely dependent on Src, could be detected in absence of the growth factor or serum. Similar to SFKs, phosphorylation of the Src substrate p130Cas was constitutively active and insensitive to EGFR inhibition. Thus, an EGFR-independent SFK signaling axis was constitutively active in these cells.

### Co-localization of SFK and E-cadherin in cell-cell junctions

**Figure 3:**
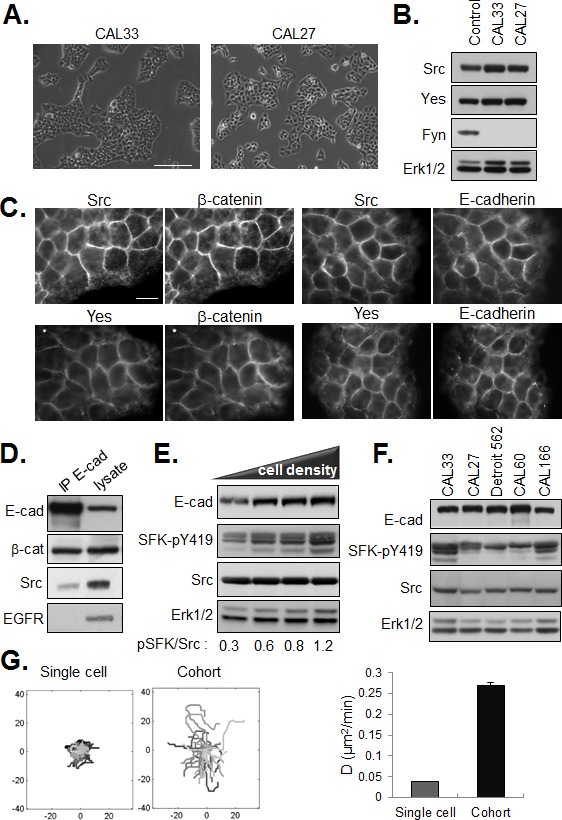
Localization of SFKs in E-cadherin based cell-cell junctions (A) Phase contrast images of CAL33 and CAL27 cells (bar=150μm). (B) Western blot of SFK members in HEK293 (control), CAL33 and CAL27 cells. ERK1/2 expression is shown as loading control. (C) Co-staining of Src or Yes with E-cadherin and β-catenin in CAL33 cells (bar=15μm). (D) Western blot analysis of Src, EGFR and β-catenin in anti-E-cadherin immunoprecipitates from CAL33 cell lysates. (E) Western blot of lysates of CAL33 cells plated at increasing density for 36h. (F) Western analysis of E-cadherin, Src and active SFK levels in the indicated HNSCC lines. (G) Migration of non-dividing single cells or cell cohorts was recorded by time lapse videomicroscopy 12 hours after plating for 24 hours. (left) Tracings from origin and (right) diffusion coefficient (D). Diffusion coefficient was calculated from MSD assuming 2D brownian movement (error bars: 95% confidence bounds estimated from linear fit).

We were intrigued by the cohesive epithelial morphology displayed by CAL33 and CAL27 cells with elevated SFK activities (Figure 3A). SFKs are known to be key regulators of E-cadherin-based cell-cell junctions and their activation is typically associated with a mesenchymal-like phenotype. HNSCC lines have been reported to express multiple SFK members, including the ubiquitously expressed Src, Yes and Fyn [18]. As shown in Figure 3B, CAL33 and CAL27 cells express Src and Yes however we were unable to detect Fyn in these cells. In both cell lines, Src and Yes were enriched in cell-cell junctions. Immunostaining revealed the co-localization of these SFK with the junctional proteins E-cadherin and β-catenin (Figure 3C). Further, co-immunoprecipitation of Src with anti E-cadherin antibodies yielded a molecular complex containing Src and E-cadherin (Figure 3D). Src has been shown to functionally cooperate with EGFR and form a heteromolecular complex when both kinases when co-expressed in fibroblasts, [19] but we failed to detect EGFR in the anti-E-cadherin immunoprecipitates, consistent with EGFR-independent SFK activation observed in our cells. Phosphorylation of SFKs increased with increasing cell density (Figure 3E and Supplemental Figure 1) suggesting that cell-cell adhesion may play an important role in this event. Indeed, E-cadherin signaling has been reported to activate c-Src at cell–cell contacts in mammary epithelial cells [20]. However, cell-cell adhesion per se was not sufficient to activate Src in CAL33 cells, as disruption and restoration of E-cadherin ligation in a Ca2+ switch assay had little effect on levels of phospho-SFK (Supplemental Figure 2). Importantly, we did not observe a decrease in E-cadherin expression coincident with SFK phosphorylation. Rather, E-cadherin levels remained elevated in dense cultures of CAL33 cells and in other HNSCC lines with elevated phospho-SFK (Figure 3E-F and not shown).

Maintenance of E-cadherin expression and a tightly cohesive epithelial phenotype in presence of robust activation of SFKs is at variance with the prevailing view that increased Src activity is associated with a disruption of E-cadherin-based adherens junctions [8]. Rather, it is consistent with a role for SFKs in the positive regulation of cohesive interactions between tumor cells. This stabilizing effect of SFKs on cell-cell junctions could exert a positive impact on the motility of adherent tumor cell cohorts. In support of this hypothesis, we observed by time lapse videomicroscopy that the migration of cells within multi-cellular clusters (high phospho-SFK levels) is increased as compared to the migration of single cells (lower phospho-SFK levels), as shown in Figure 3G.

### SFK-dependent control of cell-cell junctions

**Figure 4:**
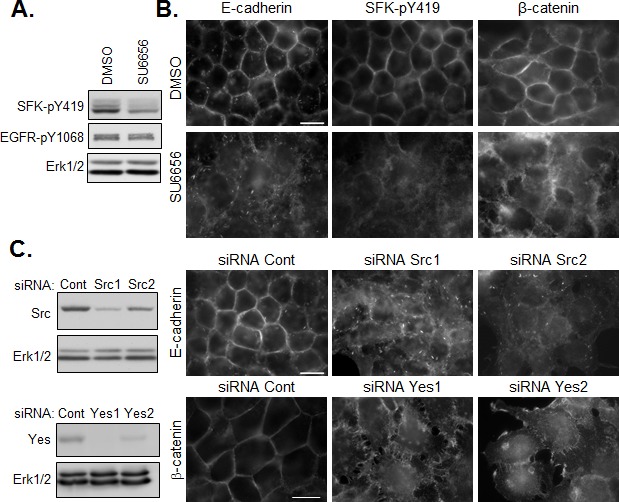
SFKs positively regulate the maintenance of E-cadherin based cell-cell junctions (A) Western analysis of SFK inhibition (SU6656, 5μM) on SFK and EGFR phosphorylation. (B) Immunostaining of active SFK (SFK-pY419), E-cadherin and β-catenin in confluent CAL33 cells treated for 12h with SU6656 (5μM) 24h after plating (bar=15μm). (C) (left) Western blot analysis of CAL33 cells 48h after transfection with control, Src- or Yes-tageting siRNA. (right) Immunofluorescent staining of E-cadherin (top) or β-catenin (bottom) in Src- or Yes-depleted CAL33 cells (bar=15μm).

**Figure 5:**
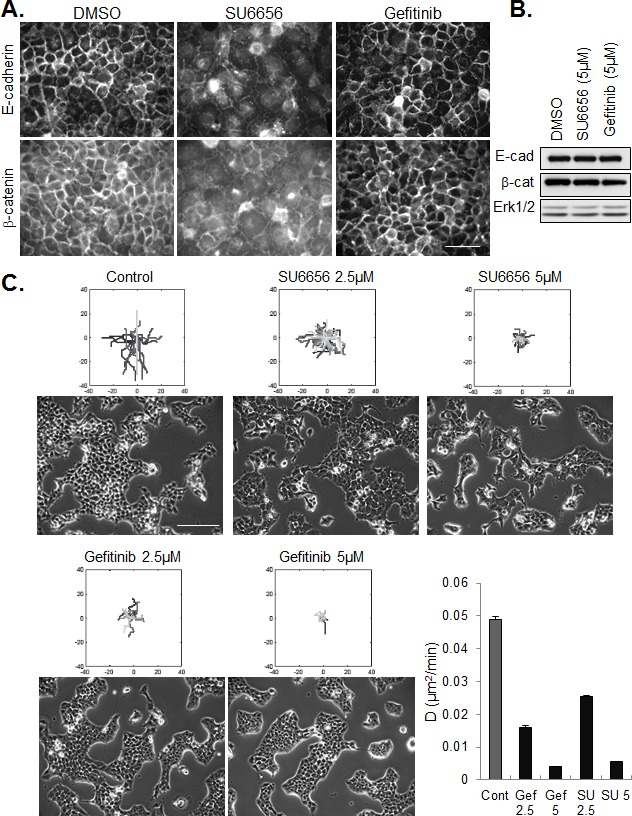
Effect of SFK and EGFR inhibitors on cell-cell junctions and collective migration (A) Immunostaining of E-cadherin and β-catenin in confluent CAL33 cells treated for 12h with SU6656 (5μM) or Gefitinib (5μM), 24h after plating (bar=50μm). (B) Western blot analysis of E-cadherin and β-catenin expression in CAL33 cells treated with the indicated inhibitor. (C) Phase contrast images (bar=200μm) of CAL33 cells treated with the indicated concentration of inhibitor are shown below tracings of cell migration after 12 hours of treatment. The dose-dependent effect of Gefinitib (Gef) and SU6656 (SU) on diffusion coefficient is represented (error bars: 95% confidence bounds estimated from linar fit).

To examine the role of active SFKs in the regulation of cell-cell junctions in HNSCC cells, we employed two loss of function approaches. First, we used the specific pharmacological inhibitor, SU6656 [21], to blunt the constitutive kinase activity of SFKs in confluent HNSCC cells. Inhibition of kinase activity was demonstrated by the decrease in phosphorylation of SFKs following SU6656 treatment of cells (Figure 4A). SFK inhibition had no effect on EGFR phosphorylation under these conditions, indicating that SFK activity did not affect basal EGFR kinase activity. As shown in Figure 4B, treatment with SU6656 reduced the accumulation of E-cadherin and β-catenin in cell-cell contacts, without affecting the total levels of the junctional proteins (Figure 5 and not shown). Disruption of intercellular junctions was accompanied by an increase in cell spreading. Similar results were observed following siRNA-mediated knockdown of Src or Yes (Figure 4C and D), suggesting that both SFKs are involved in this effect. Hence, these data support a model in which SFKs promote junctional targeting of E-cadherin and β-catenin and play a positive role in the regulation of cell-cell junctions in HNSCC cells.

Consistent with our finding that constitutive SFK activation in these cells is independent of EGFR activation, and that active SFKs stabilize intercellular contacts, treatment of tumor cells with gefitinib did not de-localize junctional staining of E-cadherin and β-catenin and the drug had no effect on their expression levels (Figure 5A and B). To the contrary, adherent groups of gefitinib-treated cells appeared more compact than untreated cell islands. Despite their opposite effects on cell-cell adhesion, inhibitors of SFKs and EGFR were similarly effective in blocking the migration of cohesive cells (Figure 5C).

### SFK activation and intercellular cohesion of cells in a 3D environment

**Figure 6:**
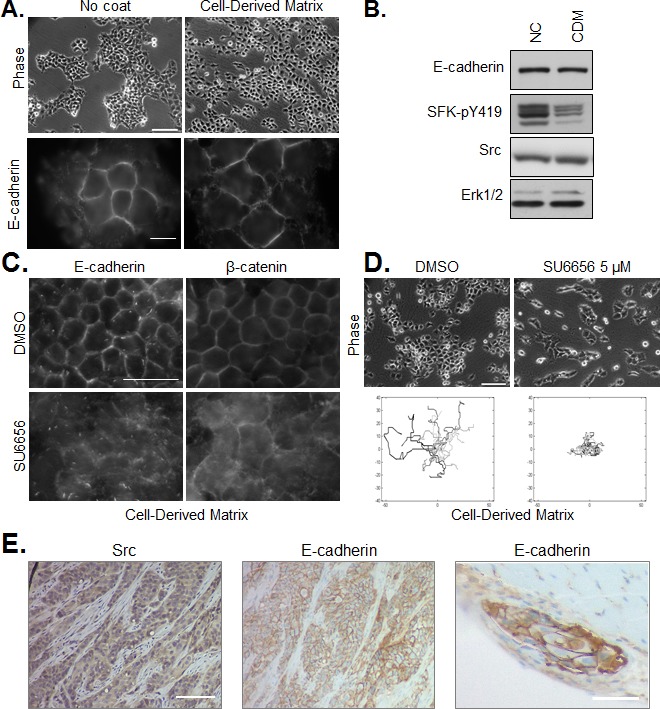
Regulation of cell cohesion and SFK on cell-derived matrix and *in vivo* (A) CAL33 cells were plated on non-coated culture plates (left) or cell-derived matrix (right). Phase contrast images (bar=150μm), and immunofluoresence staining of E-cadherin (bar=15μm) are shown. (B) Western blot of E-cadherin, SFK-pY419 and Src in CAL33 cells on non-coated culture plates (NC) or cell-derived matrix (CDM). ERK1/2 expression is shown as loading control. (C) Immunostaining of E-cadherin or β-catenin in CAL33 cells on cell-derived matrix treated for 12h with SU6656 (5μM) or DMSO control (bar=50μm). (D) Phase contrast images (bar=150μm) of CAL33 cells on cell-derived matrix treated or not with 5μM SU6656 are shown above tracings of cell migration after 12 hours of treatment. (E) Immunohistochemical staining of Src and E-cadherin in FFPE sections of CAL33-derived tumors isolated from mice (bar=200μm). An E-cadherin-positive tumor embolus in muscle tissue adjacent to the tumor mass is shown on the right panel (bar=100μm).

In order to extend the *in vivo* relevance of our results obtained in a conventional culture system, we performed experiments with tumor cells on 3D cell-derived matrices. These fibrillar matrices, produced by human telomerase-immortalized fibroblasts (TIFs) recapitulate important features (composition, topology, physical properties) of the stromal matrix in human HNSCC. As shown in Figure 6A, we observed that adhesion of tumor cells to a cell-derived matrix, as compared to tissue culture plastic, enhanced spreading. Enhanced spreading was accompanied by decrease in SFK activity (Figure 6B) and a reduction in inter-cellular cohesion, as seen in phase contrast images and immunofluorescence staining of E-cadherin. Similar to the response of cells plated on non-coated plastic, pharmacological inhibition of Src in cells plated on cell-derived matrices de-localized junctional E-cadherin, disrupted cell-cell adhesions and abrogated collective migration (Figure 6C, D).

When injected orthotopically in the floor of the mouth of nude mice, CAL33 cells rapidly form tumors with a moderately differentiated phenotype [22]. Immunohistochemical staining of Src and E-cadherin in CAL33-derived tumors, as shown in Figure 6E, revealed persisantly elevated Src and E-cadherin expression and localization of E-cadherin in cell-cell junctions of invasive tumor strands. A similar organization of cohesive E-cadherin-positive tumor cell strands was observed for CAL27 cells plated on cell-derived matrix or implanted orthotopically in mice (Supplemental Figure 3).

## DISCUSSION

### Src and cell-cell adhesion

SFK signaling is complex and it has been found to both positively and negatively affect the integrity of cell-cell interactions and cadherin function (see [9]). Whereas the proto-oncogene Src can phosphorylate adherens junction components and regulate their behavior in a tightly controlled manner leading to junction stability [23, 24], expression of oncogenic (v-Src) or constitutively active Src mutants perturbs junction assembly [11, 25, 26]. It was thus proposed that kinase activation exerts a positive supportive role at lower signal strengths and an inhibitory function at high signal strengths [20]. However, cell type and context-dependent signaling networks are additional factors that must be considered. Here we demonstrate robust SFK activity in human head and neck tumor specimens and HNSCC-derived tumor cells. Src is present in E-cadherin-based cell-cell junctions in our culture models, consistent with its junctional localization in other epithelial systems. At variance with other studies, pharmacological inhibition of SFKs in HNSCC lines or depletion of Src or Yes delocalized junctional E-cadherin and β-catenin and disrupted intercellular cohesion. These findings indicate that SFKs are involved in the stability of cadherin-dependent cell-cell contacts in at least a subset of HNSCC. Care must be used in the comparison of results from studies on cells of different origins or genetic landscapes in which the pharmacological and molecular inhibition of Src catalytic activity is achieved with different tools. In the current study, we selectively targeted SFKs by specific RNA silencing and with SU6566, a compound which is relatively specific for SFKs [21]. Other commonly employed SFK inhibitors have known additional targets and may exert effects unrelated to SFK inhibition. For example Dasatinib, a clinically relevant SFK inhibitor also inhibits BCR-ABL, c-KIT, PDGFR-α and -β and ephrin receptor kinase [27]. PP2 and PP1, have been shown to be powerful inhibitors of TGFβ receptor type I/ALK5 and type II, in addition to SFKs [28]. In the case of PP1/2, this dual inhibition (SFK/TGFβ) may yield misleading results regarding the SFK-dependent regulation of EMT. Indeed PP2 and PP1, but not SU6656, supressed TGFβ1-induced EMT in TGFβ-responsive Panc-1 pancreatic ductal adenocarcinoma cells whereas SFKs were required for basal and TGFβ1-induced cell migration [29]. It is noteworthy that the HNSCC lines we examined are unresponsive to TGFβ-induced EMT (results not shown).

Elevated SFK activity described in various epithelial malignancies is commonly associated with an aggressive phenotype characterized by the loss of an epithelial phenotype and acquisition of migratory mesenchymal features (reviewed in [8, 30]). The E-cacherin/catenin complex is an important gatekeeper in malignant progression and downregulation of E-cadherin expression is considered a hallmark of EMT [31]. Indeed, Mandal *et al.* reported that increased Src activity was associated with either quantitative and or qualitative down-regulation of E-cadherin in a majority of HNSCC cell lines and tumor specimens examined [32]. However, E-cadherin levels were high in our HNSCC lines with constitutively elevated SFK activation, and E-cadherin expression was unchanged upon inhibition of SFK activity. Beyond these *in vitro* observations, we detected strong staining of Src and membranous E-cadherin staining in our HNSCC lines grown *in vivo*, follow orthotopic injection in mice. We were unable to detect active SFK in human or grafted tumors in the present study as currently available phospho-specific antibodies did not work on FFPE tissue in our hands.

In accordance with the latter observation, a significant proportion of histopathologically invasive tumors retain an epithelial phenotype with E-cadherin expression and intact cell-cell adhesion at invading tumor fronts (reviewed in [14, 33]). Human squamous cell carcinomas typically reveal a phenotype of large invading tumor fronts, corresponding to the phenotype of collective cell movement [13]. Local environmental signals at the invasive front control the changes in E-cadherin that govern the different modes of invasion that tumor cells adopt to escape from the primary tumor and invade surrounding stroma as multicellular groups or as single cells [34].

The HNSCC lines used in our study are highly tumorigenic *in vivo* (mouse flank and orthotopic xenografts) [22, 35]. They display a moderately differentiated epithelial phenotype (*in vivo* and in culture) and they migrate more rapidly as multicellular cohorts than as individual cells.

We have confirmed our results obtained under conventional 2D culture conditions with more relevant 3D and *in vivo* models. Thus, tumor cells in contact with a fibroblast-derived fibrillar matrix in culture, or the stromal microenvironment *in vivo*, maintain E-cadherin-positive junctions and migrate in cohesive strands. Interestingly, interactions of tumor cells with the fibroblast-derived matrix, as compared to platic, relaxed intercellular cohesion and decrease the level of active SFK in tumor cells.

Head and neck tumors display considerable intratumor molecular heterogeneity and phenotypic plasticity. Using different established HNSCC lines, Basu and colleagues have isolated mesenchymal-like and epithelial-like subtypes based on E-cadherin expression levels [36, 37] that display different sensitivities to therapeutic agents used in the clinic. It would be interesting to evaluate the levels of active SFK in these mesenchymal- and epithelial-like sub-populations [36, 37]. A recent study of breast tumor epithelial cells revealed that E-cadherin positive cells can display phenotypic and molecular heterogeneity (i.e. invasive leader cells with a basal phenotype and bulk tumor cells) without undergoing a mesenchymal conversion [38]. The regulation and role of SFKs may vary among these phenotypically different tumor cell subtypes.

### Src/EGFR axis

Src kinase activation is usually placed downstream of EGFR signalling [18]. However, in the human clinical specimens we observed only a weak correlative trend between active SKFs (phosphorylated on Y419) and active EGFR (phosphorylated on Y1068) that was not observed in tumor cells (lines examined in our study). Further, SFK phosphorylation was not stimulated by EGF treatment, nor was it inhibited by selective inhibition of the EGFR. These findings are consistent with a recent study in which variable expression of activated EGFR and Src was observed, with no correlation in expression levels [39]. We were not able to investigate possible links between EGFR and Src expression/activity in the human tumors following gefitinib and irradiation since patients were not re-biopsied after treatment. In cultured tumor cells SFK activation was not only EGFR independent, it was constitutive (in absence of serum). This may result from an EGFR-independent release of growth factors or cytokines that act in an autocrine/paracrine manner to regulate motility and proliferation in these cells. Interestingly, collective cell migration was inhibited by the EGFR inhibitor gefitinib without disrupting cell-cell junctions, suggesting that Src and EGFR signalling axes may converge on a common event essential for cell motility.

Src kinases have been linked downstream to STATS 3 and 5 in HNSCC [18, 40-42]. Activated SFKs were proposed to contribute to both EGFR-dependent and -independent STAT growth pathways [43]. Interestingly, EGFR-independent phosphorylation of STAT3 has been identified as a mechanism of tumor resistance to agents targeting SFK [44]. In line with our observations, efficient STAT 3 activation mediated at least in part by Src signaling was triggered by E-cadherin engagement during cell-cell adhersion in HNSCC lines cultured in monolayer or aggregate conditions [45]. We did not examine the STAT phosphorylation status in our study or the effect of SFK inhibition on STAT phosphorylation in our HNSCC lines however inhibition of STAT-mediated signaling could contribute to the anti-proliferative/pro-apoptotic effects of SU6656 observed in our system after extended treatment and at high inhibitor concentrations (not shown) and strategies to interfere with SFKs may demonstrate therapeutic efficacy due in part to their effect on STAT signaling.

### Targeting SFK in HNSCC

Based on promising preclinical evidence on the relationship between Src and solid cancers, including HNSCC [46, 47], inhibitors such as dasatinib (BMS- 354825, SPRYCEL®, Bristol-Myers Squibb), bosutinib (SKI-606, Wyeth/Pfizer) and saracatinib (AZD0530, AstraZeneca) have been developed and are currently being used in clinical trials [6, 48]. To date however, SFK inhibitors have shown only minimal therapeutic activity in various types of solid tumors as a single agent in clinical trials. An early-phase Phase II study of saracatinib for patients with recurrent or metastatic HNSCC was closed early due to lack of efficacy [49]. The recently reported failure of this SFK inhibitor in a clinical trial involving patients with metastatic melanoma suggests a potential immune suppressive activity of this agent [50]. The immunomodulatory roles of these kinases in solid tumors that have been overlooked in immune-deficient preclinical tumor xenograft models are clearly to be evaluated in future investigations. Recently, in a comprehensive analysis of tumor grafts derived from head and neck tumor patients, dasatanib displayed *in vivo* efficacy when combined with the JAK2 inhibitor BMS911543 in tumor-bearing mice [51]. Targeting SFKs dramatically enhances the therapeutic efficacy of anti-RTK drugs (reviewed in [6]) and combinatorial regimens may prove to help in overcoming resistance to current anticancer therapies and in preventing metastatic spread.

## MATERIALS AND METHODS

### Cell culture

The human head and neck cancer cell lines, CAL33, CAL27, CAL166, CAL60 were established in the Antoine Lacassagne Cancer Centre [35] and the Detroit 562 cells derived from a metastatic pharyngeal SCC were from American Type Culture Collection (ATCC, Rockville, MD, USA). Human telomerase-immortalized fibroblasts (TIF) [52] were provided by Dr. J. Norman (Beatson Institute, Glasgow, UK). Tumor cells, including the MDAMB231 and MCF7 breast tumor cells and the SW480 and SW620 colon cancer lines (ATCC) were cultivated in DMEM (Invitrogen, Cergy Pontoise, France) containing 10% (v/v) fetal calf serum (FCS). TIFs were cultured in DMEM containing 20%FCS. Cells were routinely tested for mycoplasma by qPCR (Mycoplasma Plus, Stratagene, La Jolla, CA, USA). Cell-derived matrices produced by TIFs were prepared as described in [53].

### Patient tumor samples

HNSCC samples were obtained from patients included in the CARISSA multicenter blinded institutional review board-approved phase II trial of post-operative irradiation with cisplatin ± gefitinib (GORTEC 2004-02 - NCT00169221). Clinicopathological data have been reported [54]. Inclusion criteria required tumor samples to contain at least 50% tumor cells. For Western analyses, tumor fragments (mean=190mg) were frozen in liquid nitrogen within 15 minutes after surgery and subsequently processed for quantitative Western blotting as described [54]. The remaining mirrored tumor fragments were used for histological control and fixed in formalin for immunohistochemical analyses.

### Orthotopic xenograft model

Human HNSCC lines (CAL33 and CAL27) were implanted into the floor of the mouth of nude mice through a submandibular route, as reported in [22]. Excised tumors were fixed in formalin and embedded in paraffin for immunohistochemical staining.

### Reagents

SU6656 was from Calbiochem (La Jolla, CA, USA) and Gefitinib was kindly provided by AstraZeneca (Macclesfield, UK). EGF was from R&D Systems (Abingdon, UK).

### Antibodies

The following antibodies were used: anti-EGFR, anti-phospho-EGFR (Tyr1068), anti-phospho-Src family (Tyr416), anti-Yes, anti-Akt, anti-phospho-Akt (Ser473) and anti-p130Cas-pY410 from Cell Signalling Technology (Beverly, MA, USA); anti cortactin p80/85 (clone 4F11) and anti-phospho-cortactin (Tyr421) from Millipore (Temecula, CA USA); anti-β-catenin antibody (clone 6F9) and the monoclonal anti-phospho-ERK1/2 (Thr202/Tyr204) from Sigma-Aldrich (St. Louis, MO, USA); anti-E-cadherin and anti-p130Cas from BD Biosciences (Le Pont de Claix, France); anti-ERK1 (C-16) from Santa Cruz Biotechnology (Santa Cruz, CA, USA); anti-Src and anti-Yes from R&D Systems (Abingdon, UK). Secondary antibodies coupled to horseradish peroxidase were from Jackson Immunoresearch Labs (West Grove, PA, USA). Fluorescently-labeled secondary antibodies were purchased from Invitrogen (Cergy Pointoise, France).

### siRNA transfection

Short interfering RNAs (siRNAs) were purchased from Eurogentec (Serang, Belgium). Equal amounts of complementary RNA oligonucleotides were combined to a final concentration of 20 μM and annealed according to the protocol supplied by the manufacturer. siRNAs were transfected in cells using the Interferin reagent (Polypus-transfection SA, Illkirch, France) according to *manufacturer's protocols* and cell analyses were performed 48 hours later. The sense and antisense sequences of human Src-specific siRNA were: Src1: 5′-ACAUGAGCAAGG GGAGUUUTT-3′ and 5′-AAACUCCCCUUGCUCAUGUTT-3′; Src2: 5′-GCUGUUCGGAGGCUUCAACTT-3′ and 5′-GUUGAAGCCUCCGAACAGCTT-3′. were Yes1: 5′-UUAUGAAGCUAGAACUACATT-3′ and 5′-UGUAGUUCUAGCUUCAUAATT-3′; Yes2: 5′-GGAAGGAGAUGGAAAGUAUTT-3′ and 5′-AUACUUUCCAUCUCCUUCCTT-3′. As non-specific control, an RNA duplex designed to target the GFP transcript was used 5′-AAC AGCUGCUAGGAUUACATT-3′ and 5′-UGUAAUCCUAGCAGCUGUUTT-3′.

### Western blot analysis of human tumors and cell lines

Frozen tumor samples were homogenized and cell membranes were prepared by hypotonic lysis and high-speed centrifugation, as previously described [55]. Tumor membrane preparations, or cultured cells, were lysed with Laemmli buffer and proteins were separated by gel electrophoresis before their transfer onto Immobilion P membranes (Millipore, Bedford, MA, USA) for immunoblotting. Immune complexes were detected by enhanced chemiluminescence (GE Healthcare Amersham-Fisher Scientific, Waltham, MA, USA) and quantified using the GeneGnome Bio Imaging System (Syngene, Frederick, MD, USA). Immunoprecipitation was performed as described previously [56].

### Immunofluorescence and microscopy

For immunocytochemistry, cells plated on glass coverslips were fixed in a solution of 3% paraformaldehyde containing 3% sucrose. Cells were permeabilized with 0.2% Triton X-100. After staining, the coverslips were mounted in ProLong® Gold antifade reagent (Invitrogen). Fluorescence was observed through 40X(1.3NA), or 100X(1.3NA) oil objectives on a Zeiss inverted microscope (Axiovert 200M) equipped with a CoolSnap HQ cooled charge-coupled-device camera (Roper Scientifique, Evry, France). Phase contrast and video microscopy were performed using a 10X(0.25NA) air objective. Image acquisition was performed using the MetaMorph Imaging System (Universal Imaging Corp., Downingtown, PA). Cell migration was followed by manual tracking of non-dividing cells and analyzed using MatLab (The MathWorks), as described [57]. Stained tissue sections were visualized through 25X(0.8NA) oil objective on a Zeiss Axioplan2 microscope and images were acquired using a color ERc 5s axiocam camera (Carl Zeiss Microscopy GmbH, Jena, Germany) with Axiovision 4.8 software (Zeiss, Jena, Germany).

### Immunohistochemistry

Immunohistochemical staining of Src (polyclonal goat antibody, R&D Systems 1:100) and E-cadherin (mouse monoclonal antibody, clone 36/E-cadherin BD Transduction Laboratories, 1:100) was performed on serial 7μm deparaffinized tissue sections from formalin fixed paraffin embedded (FFPE) tumors following heat-induced Epitope retrieval. As negative control, primary antibody was omitted.

### Statistical analyses

Correlations between quantitative data sets (Src/SFK-pY419and SFK-pY419EGFR-pY1068) were determined using the non-parametric Spearman correlation test. Statistics were performed using the SPSS v15 software.

## SUPPLEMENTARY MATERIAL AND FIGURES


