# Supplementary material for: Elevated Src family kinase activity stabilizes E-cadherin-based junctions and collective movement of head and neck squamous cell carcinomas
Source: Oncotarget. 2014 Dec 26;6(10):7570–83. doi: 10.18632/oncotarget.3071 (PMC4480700; doi:10.18632/oncotarget.3071)
Supplement: Supplementary file 1 [file oncotarget-06-7570-s001.pdf]

Elevated Src family kinase activity stabilizes E-cadherin-based junctions and collective movement of head and neck squamous cell carcinomas

Supplementary Material

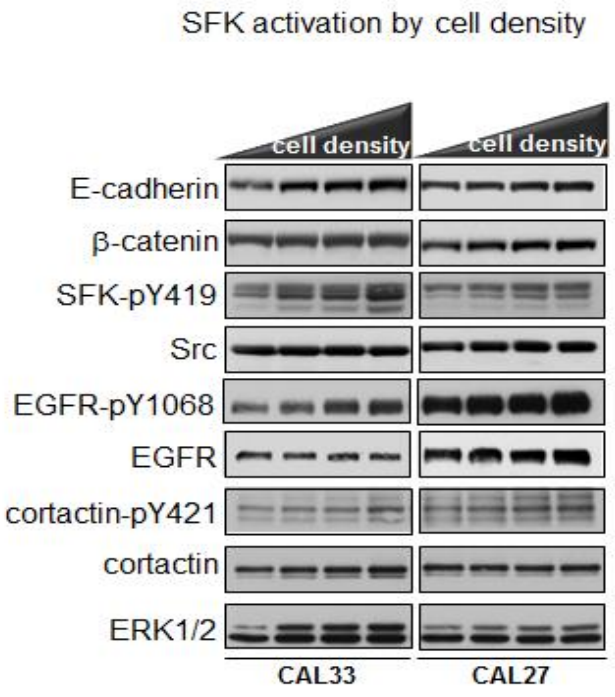

Supplemental Figure 1: Effect of cell density on SFK phosphorylation. Western blot of lysates of CAL33 and CAL27cells plated at increasing density for 36h. ERK1/2 expression is shown as loading control.

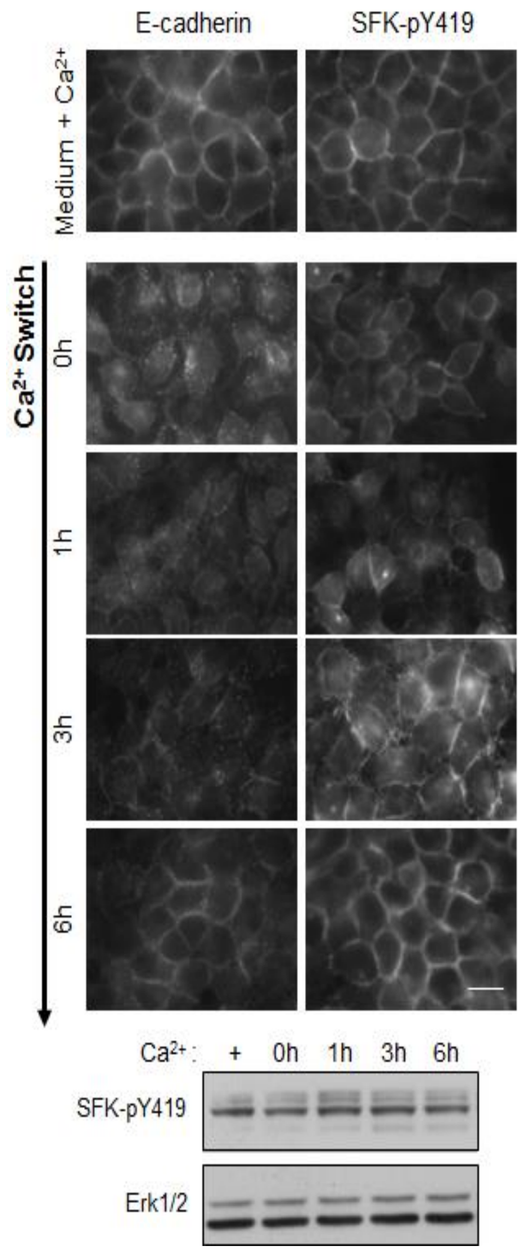

Supplemental Figure 2: Effect of cell-cell adhesion on SFK phosphorylation : Calcium switch assay. (**top**) Immunostaining of E-cadherin and phospho-SFK in CAL33 cells at the indicated times after switching cells from a Ca^{+2}-deprived to Ca^{+2} containing medium (bar=15µm). (**bottom**) Western blot analysis of SFK phosphorylation of CAL33 cell lysates following the Ca^{2+} switch. Erk1/2 is visualized as loading control.

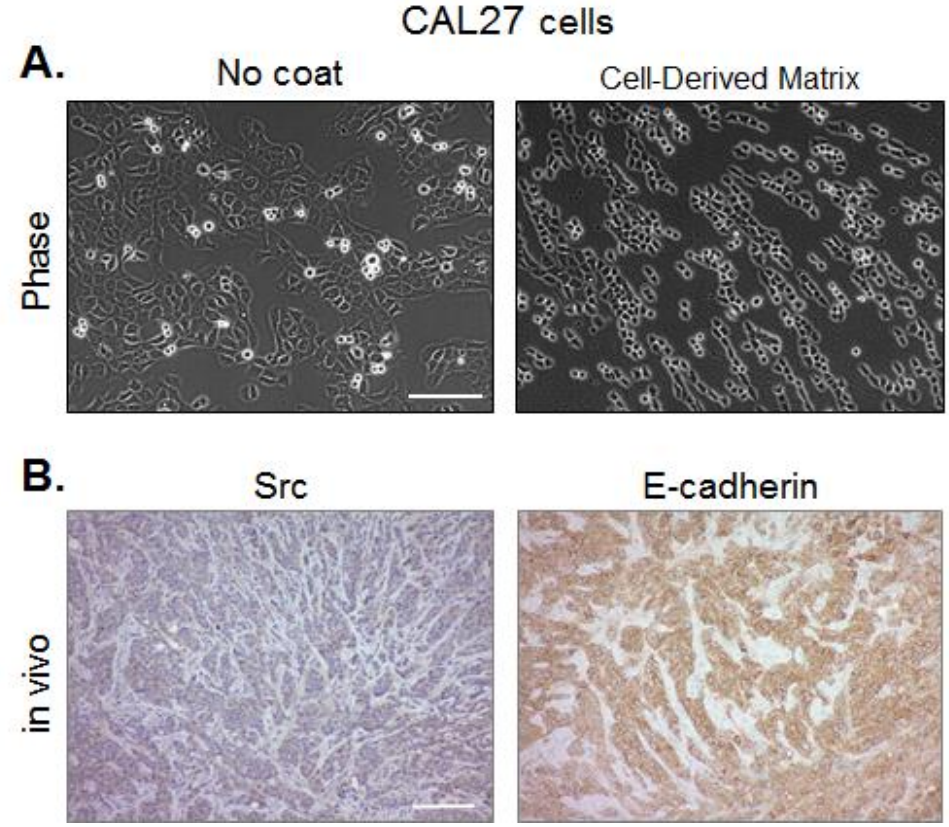

Supplemental Figure 3: Similar organization of cohesive E-cadherin-positive CAL27 cell-derived tumor cell strands on cell-derived matrix and in vivo. (**A**) CAL27 cells were plated on non-coated culture plates (left) or cell-derived matrix (right). Phase contrast images (bar=150µm) are shown. (**B**) Immunohistochemical staining of Src and E-cadherin in FFPE sections of CAL27-derived tumors isolated from mice (bar=200µm).
